# Novel sulphonamide benzoquinazolinones as dual EGFR/HER2 inhibitors, apoptosis inducers and radiosensitizers

**DOI:** 10.1080/14756366.2019.1609469

**Published:** 2019-05-10

**Authors:** Aiten M. Soliman, Ali S. Alqahtani, Mostafa Ghorab

**Affiliations:** a Department of Drug Radiation Research, National Center for Radiation Research and Technology (NCRRT), Egyptian Atomic Energy Authority (EAEA), Nasr City,Egypt;; b Department of Medicinal, Aromatic and Poisonous Plants Research Center (MAPPRC), College of Pharmacy, King Saud University, Riyadh, Saudi Arabia;; c Department of Pharmacognosy, College of Pharmacy, King Saud University, Riyadh, Saudi Arabia

**Keywords:** Benzo[g]quinazolinone, benzenesulfonamide, EGFR, HER2, apoptosis

## Abstract

A series of sulphonamide benzoquinazolinones **5–18** was synthesized and evaluated for cytotoxic activity against MDA-MB-231 cell line. The compounds showed IC_50_ ranging from 0.26 to 161.49 µM. The promising compounds were evaluated for their inhibitory profile against epidermal growth factor (EGFR) and HER2 enzymes. Compound **10** showed more potent activity on both EGFR and HER2 than erlotinib (IC_50_ 3.90 and 5.40 µM versus 6.21 and 9.42 µM). The pro-apoptotic activity of **10** was evaluated against caspase-3, Bax, B-cell lymphoma protein 2 (Bcl-2) expression levels, and cell cycle analysis. Compound **10** increased the level of caspase-3 by 10 folds, Bax level by 9 folds, decreased the level of the Bcl-2 by 0.14 and arrested the cell cycle in the G2/M phase. The radio-sensitizing activity of **10** was measured using a single dose of 8 Gy gamma radiation (IC_50_ decreased from 0.31 to 0.22 µM). Molecular docking was performed on EGFR and HER2 receptors.

## Introduction

The major challenge in cancer therapy is the induction of apoptosis through anticancer agents^1–3^. Apoptosis is a crucial process in maintaining normal tissue homeostasis in the human body, mediated by signal transduction pathways. The two major apoptotic pathways are extrinsic and intrinsic. The extrinsic pathway is induced by the trans-membrane death receptors, while the intrinsic is through mitochondrial stress caused by DNA damage and heat shock^4^. Activated caspases are the executioners of apoptosis^5^. So, more effective therapeutic strategies for better understanding of signaling pathways and molecular targets should be further provided.

Breast cancer is the world’s second leading cause of cancer-related death^6^. The overexpression of the HER2 enzyme in breast cancer is correlated with poor prognosis and drug resistance^7^. HER2 belongs to the epidermal growth factor family (EGFR), also called ErbB. It is a member of receptor tyrosine kinases (TKs) involved in signaling pathways controlling angiogenesis, cell differentiation, and proliferation^8^. The EGFR consists of a subfamily of EGFR (HER1), HER2, HER3, and HER4, that are only expressed at low levels in normal human tissues^9^. Although most patients with EGFR mutant cancers respond to therapies, the patients develop resistance after an average of one year on treatment^10^. The resistance to HER2 targeted therapies is associated with the overexpression of EGFR family enzymes^11^. It is obvious that HER family is interdependent and shows functional redundancy. The blockage of one HER receptor can be compensated by another HER family member^9^^,^^12^. The cross-linking and compensatory activities of the HER family members can provide a strong rationale for co-targeting of both EGFR and HER2 enzymes.

**Figure 1.**
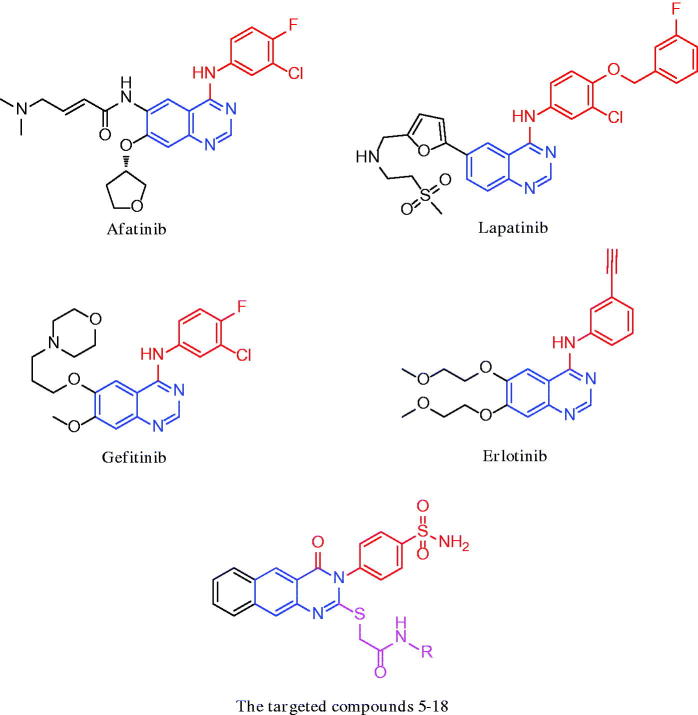
Examples of dual EGFR/HER2 inhibitors.

Molecular hybridization is a simple and effective tool to combine covalently two drug pharmacophores into a single molecule^13^. Lately, it has been observed that benzo[g]quinazoline and sulphonamides demonstrated profound growth inhibitory activity against different cancer cells and TK enzymes^14^^,^^15^. The quinazoline is a privileged scaffold that constitutes an important class of heterocyclic compounds owing to its varies pharmacological properties^16^^,^^17^. Afatinib, lapatinib, gefitinib, and erlotinib (Figure 1) are the representative drugs in this class in clinical use for targeted anticancer therapies^18–21^. The use of them has paved the way to develop new quinazoline-based molecules acting as EGFR inhibitors. Also, it is well-known that sulphonamides are strongly related to anticancer activity^22^^,^^23^. They have several targets, most of which are directly connected to oncogenesis^24^. They proved to exhibit good activity through many mechanisms as carbonic anhydrase^24^, matrix metalloprotienase^25^, NADPH reductase^26^, histone deacetylase^27^, and PI3K inhibition^28^.

In this context, we desire to exploit newer leads with tuneable anticancer activity and low toxicity^14^^,^^29^. A series of substituted benzo[g]quinazolinone benzenesulfonamide hybrids were designed, synthesized, and evaluated as dual EGFR/HER2 inhibitors. The apoptotic activity of the most potent compound was evaluated through the activation of the proteolytic caspase-3, Bax and B-cell lymphoma protein 2 (Bcl2) expression levels, cell cycle analysis, and radio-sensitizing activity. Molecular docking was carried out inside the binding site of EGFR and HER2 receptors in order to confirm their possible mechanism of action.

## Materials and methods

Melting points were uncorrected and measured on a Gallen Kamp melting point apparatus (Sanyo Gallen Kamp, UK). Precoated silica gel plates (*Kieselgel* 0.25 mm, 60 F254, Merck, Germany) were used for TLC with a developing solvent system of chloroform/methanol (7:3) and detected by the UV lamp. IR spectra were recorded using FT-IR spectrophotometer (Perkin Elmer, USA). NMR spectra were scanned on an NMR spectrophotometer (Bruker AXS Inc., Switzerland) operating at 500 MHz for ^1^H and 125.76 MHz for ^13^C. Chemical shifts are expressed in δ-values (ppm) relative to TMS as an internal standard, using DMSO-d_6_ as a solvent. Mass spectra were recorded on ISQ LT Thermo Scientific GCMS model (Massachusetts, USA). Elemental analyses were performed on a model 2400 CHNSO analyser (Perkin Elmer, USA). All the values were within ±0.4% of the theoretical values. All reagents were obtained from Sigma-Aldrich of AR grade.

### Chemistry

#### 2-[(4-Oxo-3-(4-sulfamoylphenyl)-3,4-dihydrobenzo[g]quinazolin-2-yl)thio]-N-substituted acetamide derivatives (5–18)

#### General procedure

A mixture of **4** (0.383 g, 0.001 mol) and 2-chloro-*N*-substituted acetamide derivatives (0.001 mol) in dry acetone (30 ml) and anhydrous K_2_CO_3_ (0.138 g, 0.001 mol) was stirred at room temperature for 10 h. The mixture was filtered and the product formed was crystallized from ethanol to give **5–18**.

**N-(5-Methylisoxazol-3-yl)-2-[(4-oxo-3–(4-sulfamoylphenyl)-3,4-dihydrobenzo[g]quinazolin-2-yl)thio]acetamide (5):** Yield, 68%; m.p. 292.4 °C. IR: 3403, 3305, 3190 (NH_2_, NH), 3095 (arom.), 2980, 2922 (aliph.), 1693, 1665 (2CO), 1631 (CN), 1340, 1161 (SO_2_). ^1^HNMR: 2.10 (s, 3H, CH_3_), 4.21 (s, 2H, S-CH_2_), 7.02 (s, 1H, CH isoxazole), 7.61–8.20 (m, 10H, Ar-H), 8.81 (s, 2H, SO_2_NH_2_), 9.50 (s, 1H, NH). ^13^CNMR: 18.5, 30.2, 92.7, 119.3, 119.9 (2), 124.1, 126.8 (2), 126.9, 127.7 (2), 128.0, 128.4, 130.6, 131.8, 133.7, 135.8, 145.9, 149.1, 161.2, 162.5, 169.7, 170.2. MS *m/z* (%): 521 (M^+^), 383 (100). Anal. Calcd. for C_24_H_19_N_5_O_5_S_2_ (521.08): C, 55.27; H, 3.67; N, 13.43. Found: C, 55.49; H, 3.98; N, 13.76.

**2-[(4-Oxo-3-(4-sulfamoylphenyl)-3,4-dihydrobenzo[g]quinazolin-2-yl)thio]-N-(thiazol-2-yl)acetamide (6):** Yield, 73%; m.p. 304.0 °C. IR: 3410, 3381, 3111 (NH_2_, NH), 3100 (arom.), 2970, 2881 (aliph.), 1741, 1693 (2CO), 1601 (CN), 1365, 1163 (SO_2_). ^1^HNMR: 4.20 (s, 2H, S-CH_2_), 7.01–8.20 (m, 12H, Ar-H), 8.82–8.88 (m, 3H, SO_2_NH_2_+NH). ^13^CNMR: 27.3, 113.3, 119.4, 123.3 (2), 124.4 (2), 126.6, 128.1, 128.7 (2), 129.4, 129.9, 131.0, 136.8, 137.9, 139.1 (2), 142.8, 155.4, 161.2, 167.1, 168.2. MS *m/z* (%): 523 (M^+^) (0.72), 156 (100). Anal. Calcd. for C_23_H_17_N_5_O_4_S_3_ (523.61): C, 52.76; H, 3.27; N, 13.38. Found: C, 52.98; H, 3.48; N, 13.74.

**N-(6-Ethoxybenzo[d]thiazol-2-yl)-2-[(4-oxo-3-(4-sulfamoylphenyl)-3,4-dihydrobenzo[g]quinazolin-2-yl)thio]acetamide (7):** Yield, 78%; m.p. 255.9 °C. IR: 3336, 3210, 3169 (NH_2_, NH), 3059 (arom.), 2978, 2931 (aliph.), 1680, 1678 (2CO), 1602 (CN), 1355, 1161 (SO_2_). ^1^HNMR: 1.32 (t, 3H, *J =* 10 Hz, CH_3_ ethoxy), 3.90 (s, 2H, S-CH_2_), 4.12 (q, 2H, *J =* 10.5 Hz, CH_2_ ethoxy), 6.99–8.10 (m, 13H, Ar-H), 8.82–8.86 (m, 3H, SO_2_NH_2_+NH). ^13^CNMR: 15.2, 27.3, 63.9, 105.6, 114.1, 119.3, 120.0, 123.4 (2), 126.5, 127.5 (2), 128.1, 128.6 (2), 129.2, 129.8, 130.9, 131.1, 134.0, 136.9, 139.5, 143.0, 144.4, 154.4, 156.5, 161.4, 170.3, 172.9. MS *m/z* (%): 617 (M^+^), 383 (100). Anal. Calcd. for C_29_H_23_N_5_O_5_S_3_ (617.09): C, 56.39; H, 3.75; N, 11.34. Found: C, 56.68; H, 4.09; N, 11.71.

**N-(6-Nitrobenzo[d]thiazol-2-yl)-2-[(4-oxo-3-(4-sulfamoylphenyl)-3,4-dihydrobenzo[g]quinazolin-2-yl)thio]acetamide (8):** Yield, 70%; m.p. 278.3 °C. IR: 3363, 3274, 3220 (NH_2_, NH), 3071 (arom.), 2929, 2840 (aliph.), 1710, 1695 (2CO), 1597 (CN), 1566, 1336 (NO_2_), 1336, 1165 (SO_2_). ^1^HNMR: 4.30 (s, 2H, S-CH_2_), 7.51–8.20 (m, 13H, Ar-H), 8.71 (s, 2H, SO_2_NH_2_), 8.90 (s, 1H, NH). ^13^CNMR: 31.1, 119.1, 119.3, 121.8 (2), 122.4 (2), 126.0, 127.4 (2), 128.8, 129.5 (2), 129.8 (2), 131.1 (2), 139.1 (3), 143.0 (2), 157.6 (2), 161.0, 169.2 (2). MS *m/z* (%): 618 (M^+^) (4.78), 124 (100). Anal. Calcd. for C_27_H_18_N_6_O_6_S_3_ (618.04): C, 52.42; H, 2.93; N, 13.58. Found: C, 52.78; H, 3.21; N, 13.82.

**2-[(4-Oxo-3-(4-sulfamoylphenyl)-3,4-dihydrobenzo[g]quinazolin-2-yl)thio]-N-(5-(trifluoromethyl)-1,3,4-thiadiazol-2-yl)acetamide (9):** Yield, 81%; m.p. 257.0 °C. IR: 3444, 3284, 3246 (NH_2_, NH), 3091 (arom.), 2910, 2835 (aliph.), 1715, 1695 (2CO), 1600 (CN), 1400, 1174 (SO_2_). ^1^HNMR: 4.20 (s, 2H, S-CH_2_), 7.63–8.10 (m, 10H, Ar-H), 8.81 (s, 2H, SO_2_NH_2_), 11.83 (s, 1H, NH). ^13^CNMR: 26.9, 119.4 (2), 123.5 (2), 126.5, 127.4 (2), 128.1, 128.6, 129.2 (2), 129.8 (2), 131.1, 136.9, 139.4, 145.7, 156.2 (2), 161.4 (2), 172.4. MS *m/z* (%): 592 (M^+^) (2.11), 350 (100). Anal. Calcd. for C_23_H_15_F_3_N_6_O_4_S_3_ (592.03): C, 46.62; H, 2.55; N, 14.18. Found: C, 46.30; H, 2.21; N, 13.93.

**N-(3,4-Dimethylphenyl)-2-[(4-oxo-3-(4-sulfamoylphenyl)-3,4-dihydrobenzo[g]quinazolin-2-yl)thio]acetamide (10):** Yield, 77%; m.p. 232.8 °C. IR: 3416, 3289, 3143 (NH_2_, NH), 3063 (arom.), 2948, 2842 (aliph.), 1718, 1691 (2CO), 1631 (CN), 1390, 1160 (SO_2_). ^1^HNMR: 2.15 (s, 3H, CH_3_), 2.18 (s, 3H, CH_3_), 4.12 (s, 2H, S-CH_2_), 7.03–8.21 (m, 13H, Ar-H), 8.80 (s, 2H, SO_2_NH_2_), 10.31 (s, 1H, NH). ^13^CNMR: 19.2, 20.0, 27.9, 117.2, 119.4, 120.9, 123.4 (2), 126.6, 127.4 (2), 128.1, 128.8, 129.4 (2), 129.9 (2), 130.0, 131.0, 131.7, 136.8 (2), 136.9, 137.1, 145.8, 155.4, 161.3, 165.6. MS *m/z* (%): 544 (M^+^) (1.24), 310 (100). Anal. Calcd. for C_28_H_24_N_4_O_4_S_2_ (544.12): C, 61.75; H, 4.44; N, 10.29. Found: C, 62.04; H, 4.69; N, 10.56.

**N-(2,5-Dimethylphenyl)-2-[(4-oxo-3-(4-sulfamoylphenyl)-3,4-dihydrobenzo[g]quinazolin-2-yl)thio]acetamide (11):** Yield, 78%; m.p. 279.3 °C. IR: 3388, 3269, 3212 (NH_2_, NH), 3051 (arom.), 2982, 2844 (aliph.), 1693, 1655 (2CO), 1600 (CN), 1328, 1157 (SO_2_). ^1^HNMR: 2.02 (s, 3H, CH_3_), 2.21 (s, 3H, CH_3_), 4.20 (s, 2H, S-CH_2_), 7.18–8.34 (m, 13H, Ar-H), 8.86 (s, 2H, SO_2_NH_2_), 11.16 (s, 1H, NH). ^13^CNMR: 19.3, 22.6, 30.2, 110.7, 119.2, 119.9 (2), 122.7, 124.6, 125.2 (2), 127.0, 127.4, 128.6, 128.9 (2), 129.0 (2), 129.9, 130.9, 133.8, 134.6, 135.9, 136.5, 145.2, 155.8, 161.4, 169.0. MS *m/z* (%): 544 (M^+^) (2.88), 340 (100). Anal. Calcd. for C_28_H_24_N_4_O_4_S_2_ (544.12): C, 61.75; H, 4.44; N, 10.29. Found: C, 61.62; H, 4.11; N, 10.07.

**N-(2,6-Dimethylphenyl)-2-[(4-oxo-3-(4-sulfamoylphenyl)-3,4-dihydrobenzo[g]quinazolin-2-yl)thio]acetamide (12):** Yield, 89%; m.p. 300.5 °C. IR: 3361, 3269, 3132 (NH_2_, NH), 3049 (arom.), 2972, 2871 (aliph.), 1699, 1653 (2CO), 1600 (CN), 1355, 1155 (SO_2_). ^1^HNMR: 1.78 (s, 6H, 2CH_3_), 4.22 (s, 2H, S-CH_2_), 7.54–8.32 (m, 13H, Ar-H), 8.81–8.85 (m, 3H, SO_2_NH_2_+NH). ^13^CNMR: 15.0 (2), 31.1, 119.4, 123.3 (2), 126.6 (2), 127.4 (4), 128.1, 128.8 (2), 129.4, 129.8, 131.0 (2), 136.9 (2), 139.1 (2), 142.9, 145.4, 155.0, 161.3, 166.2. MS *m/z* (%): 544 (M^+^) (1.80), 340 (100). Anal. Calcd. for C_28_H_24_N_4_O_4_S_2_ (544.12): C, 61.75; H, 4.44; N, 10.29. Found: C, 61.42; H, 4.18; N, 9.99.

**N-(2-Methyl-4-nitrophenyl)-2-[(4-oxo-3-(4-sulfamoylphenyl)-3,4-dihydrobenzo[g]quinazolin-2-yl)thio]acetamide (13):** Yield, 85%; m.p. 293.8 °C. IR: 3441, 3358, 3240 (NH_2_, NH), 3057 (arom.), 2978, 2916 (aliph.), 1697, 1664 (2CO), 1627 (CN), 1539, 1340 (NO_2_), 1357, 1161 (SO_2_). ^1^HNMR: 2.04 (s, 3H, CH_3_), 4.30 (s, 2H, S-CH_2_), 7.53–8.25 (m, 13H, Ar-H), 8.81 (s, 2H, SO_2_NH_2_), 10.03 (s, 1H, NH). ^13^CNMR: 18.3, 27.4, 105.2, 119.4, 121.2, 123.4 (2), 123.7, 123.9, 126.7 (2), 127.4, 128.2, 128.8 (2), 129.4, 129.9, 131.8, 136.8, 134.7, 139.3, 142.8, 143.8, 145.9, 155.3, 161.3, 167.0. MS *m/z* (%): 575 (M^+^) (8.50), 79 (100). Anal. Calcd. for C_27_H_21_N_5_O_6_S_2_ (575.09): C, 56.34; H, 3.68; N, 12.17. Found: C, 56.72; H, 3.77; N, 12.50.

**N-(2,4-Dioxo-1,2,3,4-tetrahydropyrimidin-5-yl)-2-[(4-oxo-3-(4-sulfamoylphenyl)-3,4-dihydrobenzo[g]quinazolin-2-yl)thio]acetamide (14):** Yield, 76%; m.p. 280.0 °C. IR: 3409, 3261, 3217 (NH_2_, NH), 3100 (arom.), 2972, 2841 (aliph.), 1741, 1701, 1681, 1653 (4CO), 1582 (CN), 1396, 1159 (SO_2_). ^1^HNMR: 4.13 (s, 2H, S-CH_2_), 5.20 (s, 1H, CH uracil), 7.51–8.22 (m, 10H, Ar-H), 8.75 (s, 2H, SO_2_NH_2_), 9.42 (s, 2H, 2NH), 10.81 (s, 1H, CONHCO). ^13^CNMR: 28.2, 78.2, 119.3, 123.9 (2), 126.6, 127.4 (2), 128.2, 128.5 (2), 129.3, 129.8, 131.0, 136.8 (2), 139.0, 142.7, 146.1, 155.2, 161.4 (2), 165.4 (2). MS *m/z* (%): 550 (M^+^) (4.50), 79 (100). Anal. Calcd. for C_24_H_18_N_6_O_6_S_2_ (550.07): C, 52.36; H, 3.30; N, 15.26. Found: C, 52.72; H, 3.67; N, 15.50.

**N-(1,3-Dimethyl-2,6-dioxo-1,2,3,6-tetrahydropyrimidin-4-yl)-2-[(4-oxo-3-(4-sulfamoylphenyl)-3,4-dihydrobenzo[g]quinazolin-2-yl)thio]acetamide (15):** Yield, 83%; m.p. 307.7 °C. IR: 3410, 3334, 3171 (NH_2_, NH), 3086 (arom.), 2963, 2831 (aliph.), 1708, 1691, 1678, 1645 (4CO), 1618 (CN), 1398, 1155 (SO_2_). ^1^HNMR: 3.41 (s, 6H, 2CH_3_), 4.13 (s, 2H, S-CH_2_), 6.58 (s, 1H, CH uracil), 7.50–8.22 (m, 10H, Ar-H), 8.81 (s, 2H, SO_2_NH_2_), 11.30 (s, 1H, NH). ^13^CNMR: 26.4, 28.6, 31.2, 73.8, 119.3, 123.4 (2), 126.7, 127.4 (2), 128.1, 128.8 (2), 129.4, 129.9, 131.0, 136.8, 139.1 (2), 142.7, 145.8, 155.1, 158.5, 161.3, 166.6, 170.1. MS *m/z* (%): 578 (M^+^) (3.42), 89 (100). Anal. Calcd. for C_26_H_22_N_6_O_6_S_2_ (578.10): C, 53.97; H, 3.83; N, 14.52. Found: C, 53.68; H, 3.59; N, 14.31.

**2-[(4-Oxo-3-(4-sulfamoylphenyl)-3,4-dihydrobenzo[g]quinazolin-2-yl)thio]-N-(pyrazin-2-yl)acetamide (16):** Yield, 81%; m.p. 205.7 °C. IR: 3429, 3325, 3246 (NH_2_, NH), 3060 (arom.), 2959, 2825 (aliph.), 1695, 1681 (2CO), 1629 (CN), 1338, 1157 (SO_2_). ^1^HNMR: 4.21 (s, 2H, S-CH_2_), 7.53–8.42 (m, 13H, Ar-H), 8.87 (s, 2H, SO_2_NH_2_), 9.24 (s, 1H, NH). ^13^CNMR: 28.9, 119.4, 123.4 (2), 126.6, 127.4 (2), 128.0, 128.8 (2), 129.4, 129.9 (2), 131.0, 136.7, 136.8 (2), 139.1, 142.7, 145.9, 149.2, 155.2, 161.3, 167.5. MS *m/z* (%): 518 (M^+^) (1.09), 129 (100). Anal. Calcd. for C_24_H_18_N_6_O_4_S_2_ (518.08): C, 55.59; H, 3.50; N, 16.21. Found: C, 55.28; H, 3.19; N, 16.03.

**N-(Naphthalene-1-yl)-2-[(4-oxo-3-(4-sulfamoylphenyl)-3,4-dihydrobenzo[g]quinazolin-2-yl)thio]acetamide (17):** Yield, 78%; m.p. 241.6 °C. IR: 3412, 3296, 3166 (NH_2_, NH), 3059 (arom.), 2981, 2860 (aliph.), 1741, 1658 (2CO), 1627 (CN), 1348, 1161 (SO_2_). ^1^HNMR: 4.33 (s, 2H, S-CH_2_), 7.45–8.24 (m, 17H, Ar-H), 8.86 (s, 2H, SO_2_NH_2_), 10.34 (s, 1H, NH). ^13^CNMR: 31.3, 108.2, 119.0, 121.2, 121.4, 122.4 (2), 123.4, 126.0 (2), 126.2, 126.5, 126.7 (2), 127.4, 128.1, 128.4 (2), 128.6, 129.5, 129.9, 131.1, 136.9 (2), 141.0 (2), 143.8, 157.9, 161.4, 166.9. MS *m/z* (%): 566 (M^+^) (7.12), 114 (100). Anal. Calcd. for C_30_H_22_N_4_O_4_S_2_ (566.11): C, 63.59; H, 3.91; N, 9.89. Found: C, 63.78; H, 4.02; N, 10.12.

**N-(9,10-Dioxo-9,10-dihydroanthracen-2-yl)-2-[(4-oxo-3-(4-sulfamoylphenyl)-3,4-dihydrobenzo[g]quinazolin-2-yl)thio]acetamide (18):** Yield, 85%; m.p. 256.7 °C. IR: 3442, 3279, 3134 (NH_2_, NH), 3061 (arom.), 2976, 2833 (aliph.), br. 1693, 1670 (4CO), 1629 (CN), 1332, 1161 (SO_2_). ^1^HNMR: 4.15 (s, 2H, S-CH_2_), 7.23–8.16 (m, 17H, Ar-H), 8.87 (s, 2H, SO_2_NH_2_), 10.43 (s, 1H, NH). ^13^CNMR: 31.1, 114.2, 119.6, 119.9 (2), 123.0, 125.6 (2), 125.7, 126.8 (2), 126.9, 127.1, 127.5, 128.8 (2), 128.9, 129.7, 130.4 (2), 131.0, 131.9 (2), 132.9, 133.3, 133.7, 136.0, 143.0, 144.1, 160.6, 160.9, 167.4, 187.1 (2). MS *m/z* (%): 646 (M^+^) (5.29), 128 (100). Anal. Calcd. for C_34_H_22_N_4_O_6_S_2_ (646.10): C, 63.15; H, 3.43; N, 8.66. Found: C, 63.41; H, 3.70; N, 9.02.

### Biological evaluation

#### MTT assay

MDA-MB-231 breast cancer cells and 184A1 normal breast cells of American Type Culture Collection were obtained from VACSERA, Egypt. Cells were cultured using Dulbecco’s Modified Eagle’s Medium (Invitrogen/Life Technologies) supplemented with 10% FBS (Hyclone), 10 µg/mL of insulin, and 1% penicillin–streptomycin. Cells were seeded in 96-well plate with cells density 1.2–1.8 × 10,000 cells/well, in a volume of 100 µL complete growth medium + 100 µL of the tested compound per well and the plate was incubated for 24 h before the MTT assay. The cell layer was rinsed with 0.25% (w/v) trypsin, 0.53 mM EDTA solution, incubated for 2 h, then the absorbance was measured at a wavelength of 570 nm^30^. IC_50_ was calculated according to the equation of Boltzmann sigmoidal concentration-response curve using Graph Pad Prism 5.

#### *In vitro* enzymatic activity assay

EGFR and HER2 kinase kits were purchased from Invitrogen. EGFR (PV3872), 0.200 mg/mL and HER2 (PV3366), 0.192 mg/mL were used. ATP solution and a kinase/peptide mixture were prepared. The plate was incubated for 1 h at room temperature. About 5 mL of the developing solution was added to each well. The plate was incubated for 1 h and then read by ELISA Reader (PerkinElmer, USA). Every experiment was repeated three times. Data represented as means ± SE from three independent experiments. Curve fitting was performed using Graph Pad Prism 5.

#### The effect on caspase-3

The Quantikine-Human active caspase-3 immunoassay **(**R&D Systems Inc., USA) is used to measure the active caspase-3 level, by adding 100 µL of the standard diluent to the zero standard wells. Cover and incubate for 2 h at room temperature. Add 100 μL of caspase-3 (active) detection antibody solution into each well except the chromogen blank. Incubate for 1 h then add 100 µL anti-rabbit IgG HRP working solution to each well and incubate for 30 min. The absorbance of each well was measured at 450 nm.

#### The effect on BAX and Bcl-2 levels

Cells were grown in RPMI 1640 containing 10% foetal bovine serum at 37 °C, stimulated with the compounds to be tested for Bax, and lysed with cell extraction buffer. This lysate was diluted in the standard diluent buffer over the range of the assay and measured for human active Bax and Bcl2 content according to the reported method^31^.

#### Analysis of the cell cycle distribution

To determine the effect of compound **10** and erlotinib on the cell cycle distribution of MDA-MB-231 cell line; cell cycle analysis was performed using the CycleTEST^™^ PLUS DNA Reagent Kit (Becton Dickinson Immunocytometry Systems, San Jose, CA, USA). Control cells with known DNA content (peripheral blood mononuclear cells) were used as a reference point for determining the DNA index for the test samples. The cells were stained with propidium iodide stain following the procedure provided by the kit then incubated at room temperature for 5 min in the dark and run on the DNA cytometer. Cell cycle distribution was calculated using CELLQUEST software (Becton Dickinson Immunocytometry Systems, San Jose, CA, USA).

#### Radiosensitizing activity

Irradiation was performed at the National Center for Radiation Research and Technology (NCRRT), Egyptian Atomic Energy Authority (EAEA), using gamma cell-40 (^137^Cs) source. Compound **10** was selected to be re-evaluated for the *in vitro* antiproliferative activity in combination with γ-irradiation using MTT assay. Cells were incubated with compound **10** in molar concentrations of 0.01, 0.1, 1.0, and 10 µM. After 2 h, cells were subjected to a single dose of 8 Gy of γ-radiation at a dose rate of 0.758 rad/s for 17.73 min, and then the anti-proliferative activity was measured 48 h after irradiation. The IC_50_ of the tested compounds was calculated after irradiation.

#### Molecular docking

Molecular modeling was performed using the Molecular Operating Environment (MOE, 10.2008) software. The protein data bank files (PDB: **1M17** and **3RCD**) were selected for this purpose. Water molecules were ignored and hydrogen atoms were added. The co-crystallized ligands in both receptors were re-docked into the active site for method standardization. The structure of compound **10** was drawn on ChemDraw and copied as smiles to MOE. Energy minimizations were performed for compound **10** using MMFF94X force field and the partial charges were calculated. Docking of **10** inside the active site of the enzyme to generate one hundred conformations. Top-scored conformation was captured by 2D and 3D images.

## Results and discussion

### Chemistry

**Scheme 1.**
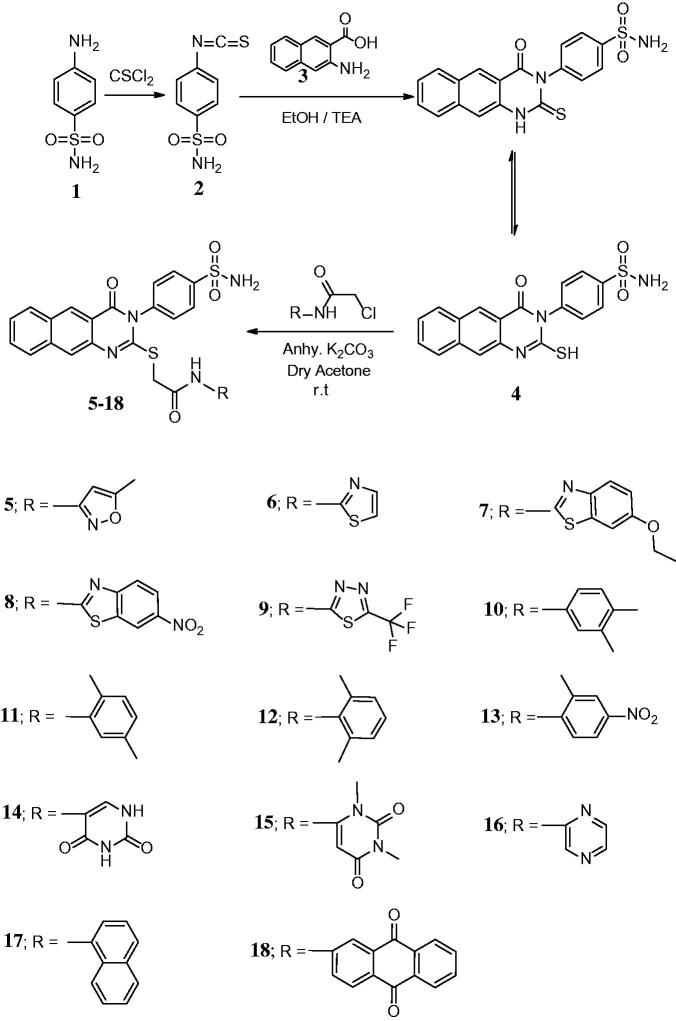
Synthesis of the benzo[g]quinazolinone derivatives **4–18**.

The synthesis of the target compounds **5–18** was described in Scheme 1. The starting compound 4-(2-mercapto-4-oxobenzo[*g*]quinazolin-3(4*H*)-yl) benzenesulfonamide **4**^29^was prepared from the reaction of 3-amino-2-naphthoic acid **3** with 4-isothiocyanatobenzenesulfonamide **2**^22^. The reaction of **4** with 2-chloro-*N*-substituted acetamide derivatives in dry acetone containing an equimolar amount of anhydrous K_2_CO_3_ yielded the appropriate *N*-(substituted)-2-[(4-oxo-3-(4-sulfamoylphenyl)-3,4-dihydrobenzo[*g*]quinazolin-2-yl)thio]acetamides **5–18** (Scheme 1). IR spectra of **5–18** revealed NH, CH aliphatic, and CO bands at their specified regions. ^1^H-NMR spectra of **5–18** revealed two singlets at 3.90–4.33 ppm attributed to the CH_2_ and 8.81–11.83 ppm attributed to the NH protons and the disappearance of SH singlet at 2.01 ppm of **4**. ^13^C-NMR spectra of **5–18** exhibited two downfield signals attributed to the C–S and CO carbons. The ^1^HNMR spectrum of **5** revealed two singlets at 2.10 and 7.02 ppm corresponding to the CH_3_ and CH isoxazole. ^13^C-NMR of **5** showed an up-field signal at 18.5 ppm due to CH_3_. ^1^HNMR of **7** showed triplet at 1.32 ppm and quartet at 4.12 ppm due to the ethoxy group. ^13^C-NMR of **7** showed two up-field signals at 15.2 and 63.9 ppm due to the ethoxy carbons. IR of **8** revealed the NO_2_ peaks at 1566 and 1336 cm^−1^. ^13^C-NMR of **9** showed a signal at 119.4 ppm for the CF_3_ carbon. ^1^HNMR of **10–12** revealed singlets at the range of 1.78–2.21 ppm due to the 2CH_3_ and ^13^C-NMR showed two signals in the range of 15.0–22.6 ppm. IR of **13** showed the NO_2_ peaks at 1539 and 1340 cm^−1^. ^1^HNMR of **13** revealed a singlet at 2.04 ppm for the CH_3_. IR of **14** and **15** showed 4 CO peaks in their specified regions. ^1^HNMR of **14** revealed two singlets at 5.20 and 10.81 ppm corresponding to the CH uracil and CONHCO, respectively. ^1^HNMR of **15** revealed two singlets at 3.41 and 6.58 ppm due to 2CH_3_ and CH uracil, respectively. ^13^C-NMR of **15** showed two signals at 28.6 and 31.2 ppm for the 2CH_3_. ^13^C-NMR of **18** revealed a signal at 187.1 due to the 2CO of anthraquinone.

### Biological evaluation

#### *In vitro* cytotoxic activity against MDA-MB-231 cell line

The *in vitro* cytotoxicity of the targeted compounds **5–18** was measured using MTT assay against human breast cancer cell line (*MDA-MB-231*), and erlotinib was used as the reference drug. Table 1 indicates that compounds **5–18** showed IC_50_ values in the range of 0.26–161.49 µM, in comparison to erlotinib (IC_50_= 0.48 µM). Compounds **10**, **11**, **13**, **14**, **16**, and **18** were more active than the reference drug, with IC_50_ values in the range of 0.26–0.40 µM. The 9,10-dioxo-9,10-dihydroanthracene derivative **18** was the most active followed by the 2,5-dimethyl phenyl **11,** the 3,4-dimethyl phenyl **10**, the pyrazinyl **16**, the 2,4-dioxopyrimidinyl **14**, and the 2-methyl-4-nitrophenyl derivative **13**. The EGFR inhibitory profile of the synthesized compounds **5–18** was measured and reported in Table 1. The results showed that the tested compounds exhibited inhibitory activity towards EGFR, ranging from 72.90% to 8.71%. The most cytotoxic compounds showed the highest inhibitory profile. Compound **14** showed the highest percentage inhibition followed by **11, 10,** erlotinib, **13**, and **18** (percentage inhibition ranging from 72.90% to 67.26%).

**Table 1.** The cytotoxic activity and percentage inhibition of compounds **5–18** on EGFR against MDA-MB-231 breast cancer cell line.

Compound no.	IC_50_ against MDA-MB-231 (µM)*	% Inhibition of EGFR
**5**	2.47 ± 0.08	42.00
**6**	23.24 ± 1.88	18.13
**7**	26.47 ± 2.14	25.98
**8**	2.91 ± 0.03	54.61
**9**	2.19 ± 0.05	52.49
**10**	0.31 ± 0.01	69.04
**11**	0.28 ± 0.01	70.34
**12**	2.19 ± 0.09	41.86
**13**	0.40 ± 0.01	67.36
**14**	0.37 ± 0.01	72.90
**15**	21.80 ± 1.63	21.45
**16**	0.32 ± 0.01	59.66
**17**	161.49 ± 4.69	8.71
**18**	0.26 ± 0.01	67.26
Erlotinib	0.48 ± 0.01	68.30

*The values represent the mean ± SE of three independent experiments.

#### EGFR and HER2 inhibition

The IC_50_ values of the compounds showing the highest percentage inhibition towards EGFR were determined. Compounds **10**, **11**, **13**, **14**, and **18** were screened on both EGFR and HER2 enzymes in reference to erlotinib. From the results in Table 2, we can conclude that all the tested compounds together with erlotinib have better inhibitory activity and lower IC_50_ on EGFR than HER-2 enzyme except for compound **18** (IC_50_ ranges from 2.55 to 10.20 µM towards EGFR versus 3.20–31.31 µM towards HER2). The 3,4-dimethyl phenyl derivative **10** was more potent than erlotinib on both EGFR and HER2 (IC_50_ 3.90 and 5.40 µM versus 6.21 and 9.42 µM, respectively). Compound **11** was the most potent towards EGFR (IC_50_ 2.55 µM), while compound **18** was the most potent towards HER2 (IC_50_ 3.20 µM).

**Table 2.** IC_50_ of compounds **10**, **11**, **13**, **14**, and **18** against EGFR and HER2 enzymes.

Compound no.	EGFR IC_50_ (µM)*	HER2 IC_50_ (µM)*
**10**	3.90 ± 0.03	5.40 ± 0.12
**11**	2.55 ± 0.17	31.31 ± 0.31
**13**	10.20 ± 0.10	13.01 ± 0.09
**14**	4.11 ± 0.02	26.03 ± 0.22
**18**	9.66 ± 0.08	3.20 ± 0.04
Erlotinib	6.21 ± 0.31	9.42 ± 0.21

*The values represent the mean ± SE of three independent experiments.

#### Activation of caspase-3

Caspase-3 is a member of the cysteine-aspartic acid protease family that plays a crucial role in apoptosis^32^. It is an inactive proenzyme converted to the active form through caspases 8, 9, and 10^33^. Caspase-3 is activated in the apoptotic cell by both extrinsic (death ligand) and intrinsic (mitochondrial) pathways^34^ by cleaving multiple proteins in the cells leading to cell death^35^. The effect of compound **10** on caspase-3 was evaluated in reference to erlotinib. Compound **10** showed an increase in the level of the active caspase 3 by 10 folds compared to the control cells. While erlotinib increases the level of caspase 3 by 9 folds (Table 3).

**Table 3.** The effect of compound **10** on the level of caspase-3.

Compound no.	Caspase 3 (Pg/mL)	Folds
**10**	545.7	10.25
Erlotinib	480.1	9.02
Control	53.2	–

#### Effects on Bcl-2 family proteins

The Bcl-2 family plays a central role in tumour progression or inhibition of mitochondrial intrinsic apoptotic pathway^36^. The pro-apoptotic Bax is essential for cell apoptosis. However, the anti-apoptotic Bcl-2 overexpression enhances cell survival by suppressing apoptosis^37^. Thus, the balance between these two different proteins determines the cell fate^38^^,^^39^. Increments in the Bax/Bcl2 ratio trigger a cascade of caspases that leads to the activation of caspase 3; the apoptosis executioner^40^. In this study, MDA-MB-231 breast cells were treated with compound **10** and their effect on the expression levels of Bcl2, and Bax were illustrated in Table 4.

**Table 4.** The effect of compound **10** on Bax/Bcl2 expression levels.

Compound no.	Bax (Folds)	Bcl2 (Folds)
**10**	9.37193	0.13794
Erlotinib	11.0418	0.07182

Compound **10** and erlotinib boosted the level of the pro-apoptotic protein Bax by 9 and 11 folds, respectively, compared to the control cells. On the other hand, they markedly reduced the levels of the anti-apoptotic proteins Bcl2 by 0.14 and 0.07 folds, respectively. The results showed that both compound **10** and erlotinib markedly boosted the Bax level and down-regulated Bcl2 level proving their pro-apoptotic effect.

#### Cell cycle analysis

**Figure 2.**
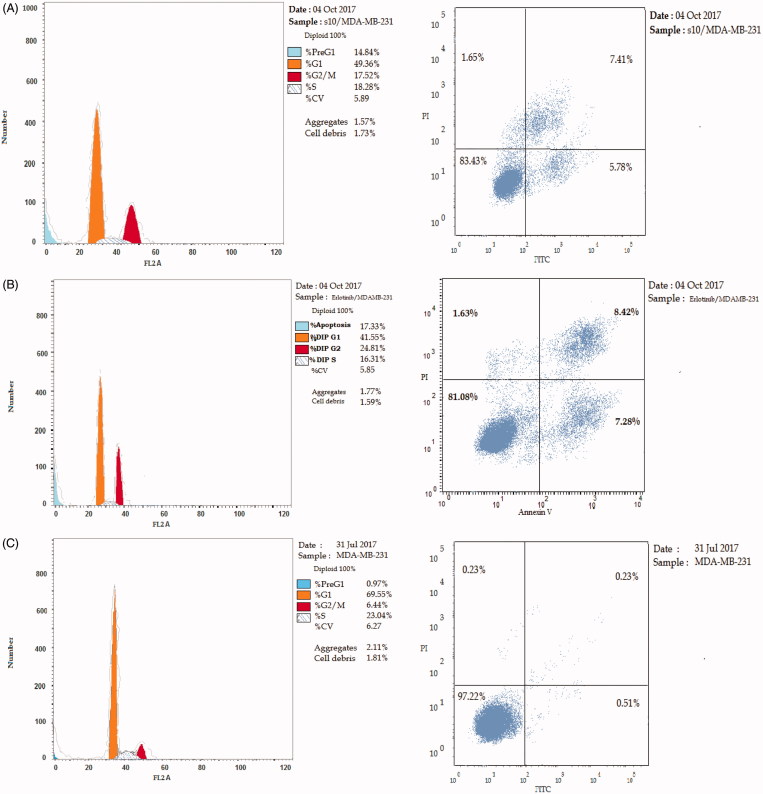
The effect of inhibitors on the phases of the cell cycle (A) compound **10**, (B) erlotinib, and (C) control MDA-MB-231 cells.

Cell cycle progression is responsible for normal cell growth and proliferation. DNA damage can lead to either DNA repair or cell death through apoptosis. The condition of the cells is assessed at certain checkpoints that act as control mechanisms to ensure the proper cell division. Cell cycle checkpoints are the G1 (restriction), the S (metaphase), and the G2/M^41^. The role of anticancer agents is to stop the cell division at these checkpoints. Treatment with the anticancer agents can determine at which phase apoptosis occurs in the cell cycle. In this study, MDA-MB-231 cells were treated with compound **10** at its IC_50_. The results in Table 5 indicate that compound **10** arrested the cell cycle at the G2/M phase when compared to the untreated control (17.52% and 6.44%, respectively; Figure 2(A,C)). While erlotinib arrested the cell cycle at the G2/M phase by 24.81% (Figure 2(B)). Also, the cell population in G1 and S phases decreases after treatment (49.36% and 18.28% versus 69.55% and 23.04%, respectively) in case of compound **10** compared to control. While in the case of erlotinib, the cell population in G1 and S phases markedly decreases after treatment to (41.55% and 16.31%, respectively). These results reveal that in MDA-MB-231 cells, cell cycle arrest occurs in the G2/M phase in the case of compound **10** and erlotinib.

**Table 5.** The effect of compound **10** and erlotinib on the phases of cell cycle.

Compound no.	%G0-G1	%S	%G2-M	%Apoptosis
**10**	49.36	18.28	17.52	14.84
Erlotinib	41.55	16.31	24.81	17.33
Control	69.55	23.04	6.44	0.97

#### Cytotoxicity against normal breast cells

The cytotoxicity of compound **10** compared to erlotinib was measured against 184A1 normal breast cells using MTT assay in order to determine the relative safety of compound **10** on normal tissues. Compound **10** and erlotinib showed mild cytotoxic effect with an IC_50_ of 84.5 and 101.9 µM, respectively (Table 6).

**Table 6.** The cytotoxicity of compound **10** and erlotinib on 184A1 normal breast cells

Compound no.	IC_50_ (µM)
**10**	84.50 ± 0.72
Erlotinib	101.9 ± 3.55

#### Radiosensitizing evaluation

Most cancer patients receive radiation therapy during the course of treatment. Gamma rays are high energy radiation used in therapy to shrink tumours and kill malignant cells by damaging their DNA either directly or indirectly through free radicals formation. The major drawback of radiation therapy is that they cannot differentiate between normal and cancerous cells. So, the use of radiotherapy and selective chemotherapy are required in order to eliminate normal tissue damage^42^.

The cytotoxicity of compound **10** was measured on MDA-MB-231 cell line before and after being subjected to a single dose of 8 Gy γ-irradiation. The ability of compound **10** to enhance the cell-killing effect of γ-irradiation was examined. The results showed that compound **10** is able to sensitize the cancer cells to the lethal effects of gamma radiation (Table 7).

**Table 7.** IC_50_ of compound **10** on MDA-MB-231 cells before and after being subjected to a single dose of 8 Gy γ-radiation.

Compound no.	IC_50_ before Irradiation (µM)	IC_50_ after Irradiation (µM)
**10**	0.31 ± 0.01	0.22 ± 0.03

#### Molecular docking

Molecular docking was performed using MOE 10.2008 inside the active site of EGFR (PDB ID: **1M17**)^43^ and HER2 receptors (PDB ID: **3RCD**)^44^. In order to rationalize the biological results and to gain insight into the SAR of the target compounds, an attempt to interpret the observed enzymatic activities of the tested compounds on the basis of the ligand-protein interactions was done. The enzymatic activity of EGFR and HER2 inhibitors depends on the ability of the compound to properly dock into the binding site and to establish interactions with the key amino acids. Accordingly, the active compound in this study should attain the same binding mode observed for the ligand.

#### Docking on EGFR

**Figure 3.**
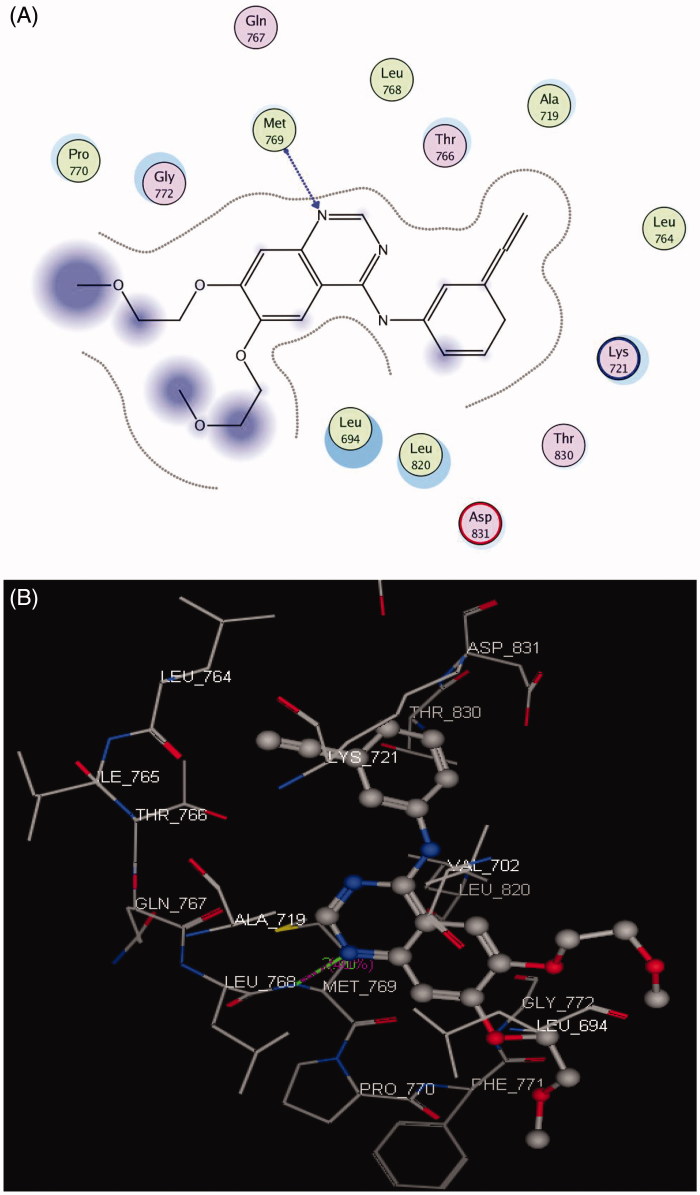
2 D and 3 D ligand interactions of erlotinib inside the active site of **1M17**.

**Figure 4.**
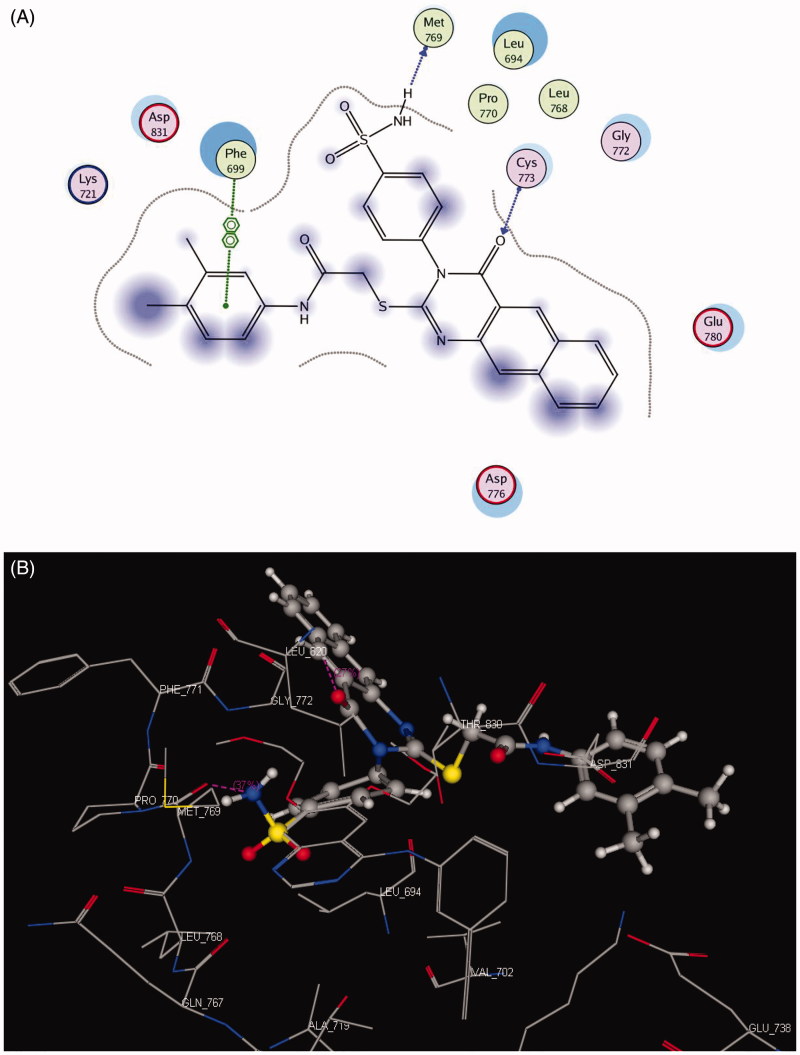
2 D and 3 D interaction maps of compound **10** inside the active site of **1M17**.

The EGFR catalytic domain consists of an N-terminal lobe, which consists mainly of one α-helix and C-helix. The C-terminal lobe is mainly α-helical, and a short strand termed the hinge region connects the two lobes^45^. The *N*-(3-ethynylphenyl)-6,7-bis(2-methoxyethoxy)quinazolin-4-amine (erlotinib) is the co-crystallized ligand inside the EGFR receptor^46^. Erlotinib was located well in the ATP pocket and interacts with Met 769 by a hydrogen bond of 2.70 A° length, and hydrophobic interactions with Leu 694, Leu 820, Lys 721, and Thr 766 (hinge region; Figure 3). Compound **10** was docked in the active site of the enzyme and bound in the same manner as the ligand. Compound **10** binds with energy score (*S* = −9.88 Kcal/mol) and interact with the active site through Met 769 by a hydrogen bond of 0.85 A°, Cys 773 with the CO of quinazolinone and Phe 699 with the phenyl ring of the acetamide through a π–π interaction (Figure 4, Table 8).

**Table 8.** Docking results of compound **10** inside **1M17** and **3RCD** active sites.

Receptor	Compound	Energy score (S) (Kcal/mol)	Amino acids	Interacting groups	Length (Å)
1M17	Erlotinib	−9.82	Met 769	N-1 of quinazolinone	2.70
	**10**	−9.88	Met 769	NH_2_ of sulphonamide	0.85
			Cys 773	CO of quinazolinone	1.91
			Phe 699	Ph of acetamide	4.23
3RCD	TAK-285	−9.70	Met 801	N-1 of pyrrolopyrimidine	2.18
	**10**	−9.71	Met 801	SO_2_ of sulphonamide	3.15
			Thr 862	CO of acetamide	1.97
			Asp 863	CO of acetamide	2.73
			Lys 753	N-1 of quinazolinone	1.87

#### Docking on HER2

**Figure 5.**
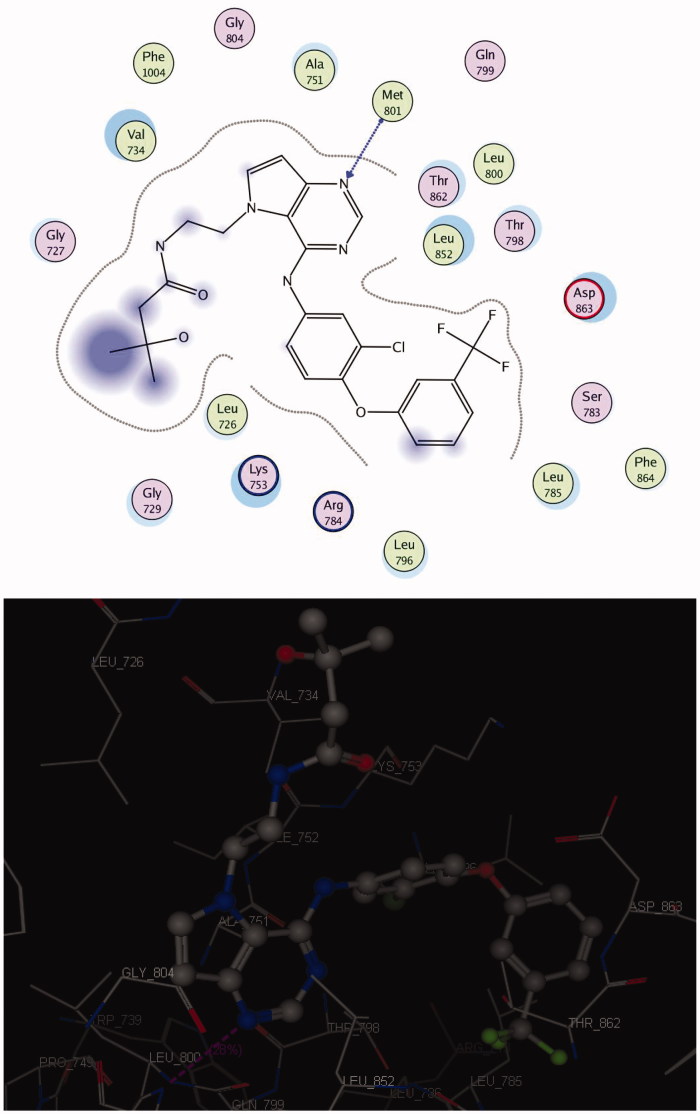
2 D and 3 D interaction maps of TAK-285 inside the active site of **3RCD**.

**Figure 6.**
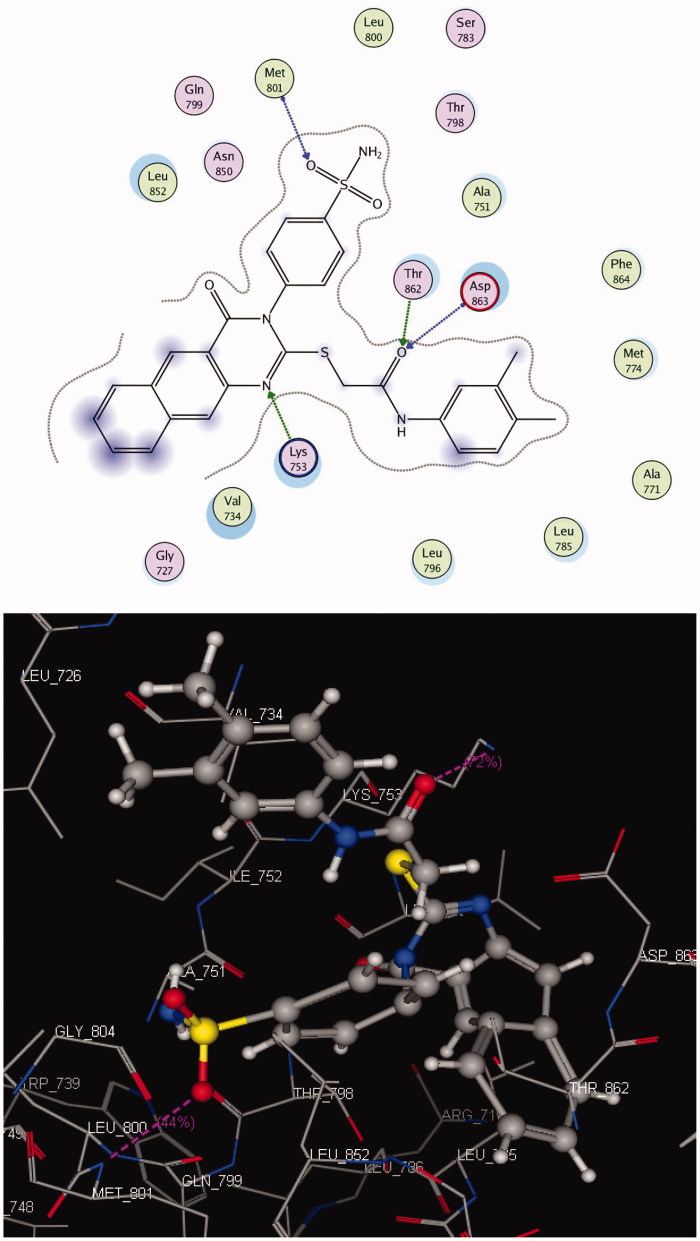
2 D and 3 D interaction maps of **10** inside the active site of **3RCD**.

The crystal structure of HER2 complexed with TAK-285 (PDB ID: **3RCD**) showed that Ala 751, Leu 800, Met 801, Leu 852, and Asp 863 are the key amino acids. The X-ray co-crystallized structure of TAK-285 with HER2 demonstrated that it binds to the ATP pocket through an H-bond with Met 801 and to the hinge region by a series of hydrophobic interactions with Leu 852, Leu 726, Phe 1004, Thr 798, Thr 862, and Leu 785^47^ (Figure 5). Compound **10** pursued the similar binding pattern in HER2 with Met 801 by the SO_2_ of the sulphonamide group, Thr 862 and Asp 863 by the CO of the acetamide and Lys 753 with the N-1 of quinazolinone (Figure 6, Table 8).

## Conclusion

An array of new 3,4-dihydrobenzo[g]quinazolinone derivatives containing sulphonamide moiety was designed, synthesized, and evaluated for their cytotoxic effect on MDA-MB-231 breast cancer cell line. The tested compounds showed IC_50_ values ranging from 0.26 to 161.49 µM on MDA-MB-231. The new compounds were tested for their inhibitory profile against EGFR and HER2 enzymes. The 3,4-dimethyl phenyl derivative **10** was more potent than erlotinib on both EGFR and HER2 (IC_50_ 3.90 and 5.40 µM versus 6.21 and 9.42 µM, respectively). The 2,5-dimethyl phenyl derivative **11** was the most potent towards EGFR, while the anthraquinone derivative **18** was the most potent towards HER2. Compound **10** was evaluated as an apoptosis inducer through the activation of the proteolytic caspase-3, Bax and Bcl-2 expression levels, and cell cycle analysis. It was found that compound **10** increases the level of caspase-3 by 10 folds, Bax level by 9 folds, decreases the level of Bcl-2 by 0.14 folds and arrested the cell cycle in the G2/M phase. The radiosensitizing activity of **10** was measured on MDA-MB-231 cell line after being irradiated by a single dose of 8 Gy. IC_50_ decreased from 0.31 to 0.22 µM after being irradiated. Docking of **10** inside the active site of EGFR and HER2 receptors revealed that it binds in the same manner as that of the co-crystallized ligand.
